# High efficiency and dynamic modulation of nonlinear metasurfaces

**DOI:** 10.1038/s41377-024-01592-1

**Published:** 2024-09-10

**Authors:** Ruizhe Zhao, Lingling Huang

**Affiliations:** Beijing Engineering Research Center of Mixed Reality and Advanced Display, MIIT Key Laboratory of Photoelectronic Imaging Technology and System of Ministry of Education of China, School of Optics and Photonics, Beijing Institute of Technology, Beijing, 100081 China

**Keywords:** High-harmonic generation, Nanophotonics and plasmonics

## Abstract

Metasurfaces have facilitated numerous innovative applications in the scope of nonlinear optics. However, dynamically tuning the nonlinear response at the pixel level is very challenging. Recent work proposed a novel method to electrically manipulate the local amplitude and phase of third-harmonics generation (THG) by integrating the giant nonlinear responses resulting from intersubband transitions of multiple quantum wells (MQW) with plasmonic nano-resonator. The demonstrated method may pave the way to realize nonlinear optical elements with versatile functionalities by electrically tuning and promoting the advancements of innovative applications such as lidar, 3D displays, optical encryption, optical computing, and so on.

## Abstract (web summary)

High efficiency and dynamic modulation of third-harmonics generation (THG) is realized by integrating the giant nonlinear responses resulted from intersubband transitions of multiple quantum well (MQW) with plasmonic nano-resonator.

## Graphical abstract

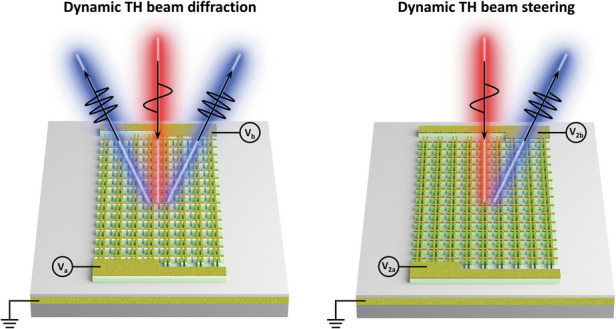

The discovery of laser provides the feasibility of generating high-intensity optical fields, which promotes the advancements of nonlinear optics and leads to various novel phenomena, including high-harmonics generation, frequency mixing, optical switching, and so on^1^. The generated high-order harmonics can be treated as new light sources and are highly desired in lithography, imaging, microscopy, and quantum optics. The nonlinear processes are closely related to the power of incident light, nonlinear polarizability of materials as well as phase-matching conditions. However, the rigorous phase-matching conditions and cumbersome setup may limit the efficiency and modulation of the nonlinear processes. Spatially tailoring the nonlinear material based on quasi-phase matching can enhance the efficiencies of second-harmonics generation (SHG) and third-harmonics generation (THG) owing to the additional momentum provided by the periodic structure. However, pixelated modulation of fundamental properties of light in the nonlinear frequency generation region is still insufficient and facing great challenges^2^.

Metasurfaces, composed of two-dimensional arrays of engineered subwavelength meta-atoms, have emerged as a transformative technology in the field of optics. The abundant design freedoms provided by the meta-atoms as well as various mechanisms of wavefront modulations, make the metasurfaces exhibit unprecedented abilities of manipulating the multi-dimensional properties of output wavefront for both linear and nonlinear optics^3,4^. For example, a nonlinear geometric phase is demonstrated for continuously controlling the local phase of generated harmonics by considering the selection rules for frequency conversion processes as well as the spatial symmetry of meta-atoms^5^. Spin and wavelength multiplexed nonlinear holography is realized based on a plasmonic metasurface composed of split ring resonators with different orientation angles^6^. Meanwhile, diatomic and quad-atom metasurfaces consist of gold meta-atoms with threefold rotational symmetry and are designed to realize nonlinear vectorial holography^7,8^. However, the high dissipative loss and low optical damage threshold of plasmonic metasurfaces limit the obtainable nonlinear conversion efficiency of nonlinear optical processes.

To relieve such limitations, high-index dielectric nano-resonators supporting both electric and magnetic resonances have become candidates for enhancing the efficiency of nonlinear optical processes with high damage thresholds. Silicon metasurfaces are utilized to realize nonlinear wavefront shaping, including nonlinear beam deflection, nonlinear vortex beam generation, as well as nonlinear holography multiplexing^9–11^. Meanwhile, the optical resonant mode with high-quality factors, including Fano resonance^12^, quasi-BIC resonance^13^, and electromagnetically induced transparency (EIT)^14^, can be utilized to acquire strong nonlinear effects based on the enhancement of light–matter interaction at the subwavelength scale. Furthermore, the strong coupling of plasmonic resonances with intersubband transitions of MQW can facilitate the generation of giant nonlinear response^15^. An electrically tunable nonlinear polaritonic metasurface has been implemented for simultaneously tuning the spectral, magnitude, and phase of SHG. Such functionality is attributed to the intersubband nonlinearities of MQW that can be modified and spectrally tuned by applying different voltages. However, the previously achieved maximum phase change of the harmonic signal by using electric tuning is limited to 135°, allowing only limited dynamic beam manipulation^16^.

In a recent work^17^ published in *Light: Science & Applications*, Park et al. demonstrate a method of electrically tuning the wavefront of THG. An MQW combined with a plasmonic nano-resonator is chosen as the meta-atom to construct the reconfigurable nonlinear metasurface. By leveraging the intersubband Stark tuning effect, the magnitude and phase of the third-order nonlinear response of the MQW structure can be electrically tuned. The thickness of the MQW layer is optimized for acquiring giant third-order nonlinear response. Meanwhile, the plasmonic nano-resonator is elaborately designed to guarantee the out-coupling of the third harmonic (TH) signal generated in the MQW region to free space. With the range of local phase tuning over 180°, dynamic TH beam diffraction tuning and TH beam steering with 86% suppression of the zeroth order beam as shown in Fig. 1 are successfully obtained.

**Fig. 1:**
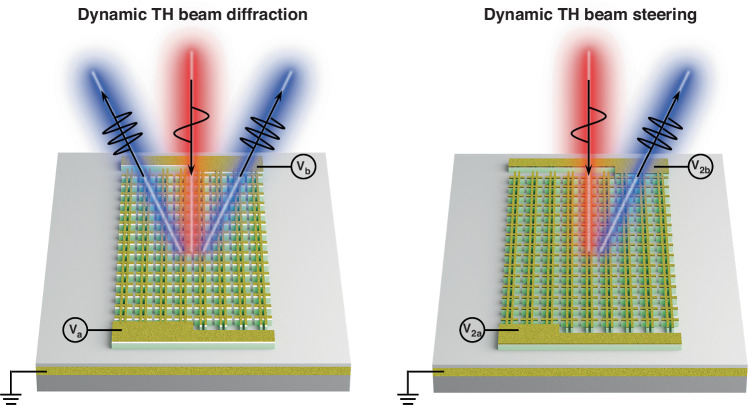
Schematic illustrations of demonstrated intersubband polaritonic metasurface. By applying a modest bias voltage (−3 to +3 V), the local amplitude and phase of THG can be electrically tuned. Such reconfigurable characteristic leads to the realization of dynamic TH beam diffraction as well as dynamic TH beam steering

Reconfigurable characteristics driven by external stimuli can significantly expand the utility of both linear and nonlinear metasurfaces. By integrating the dielectric meta-atoms with a liquid crystal layer, piezoelectric micro-electromechanical system (MEMS) or illuminating the metasurfaces with structured light generated from digital micromirror device (DMD), various reconfigurable functionalities have been successfully demonstrated in linear optics^18–20^. This demonstrated method may also be applied to nonlinear optics and provide the possibilities for realizing dynamic modulation of amplitude, phase, and polarization of generated nonlinear processes within ultra-compact optical systems.
